# The efficacy of extracorporeal shock wave therapy for knee osteoarthritis : an umbrella review

**DOI:** 10.1097/JS9.0000000000001116

**Published:** 2024-01-18

**Authors:** Peiyuan Tang, Ting Wen, Wenhao Lu, Hongfu Jin, Linyuan Pan, Hengzhen Li, Biyun Zeng, Yang Zhou, Wenfeng Xiao, Yusheng Li

**Affiliations:** Departments ofa Orthopedics; b Clinical Nursing; c National Clinical Research Center for Geriatric Disorders, Xiangya Hospital, Central South University, Changsha, China

**Keywords:** ESWT, Extracorporeal shock wave therapy, knee osteoarthritis, umbrella review

## Abstract

**Background:**

An umbrella review was conducted to compare the effectiveness of extracorporeal shock wave therapy (ESWT) versus non-ESWT in the treatment of knee osteoarthritis (KOA).

**Materials and methods:**

Three databases including PubMed, Embase and Web of science were searched up to September 2023. Literature screening, quality evaluation, and data extraction were performed according to inclusion and exclusion criteria. Meta-analysis of outcome indicators was performed using Revman 5.4 software.

**Results:**

A total of eight meta-analysis were included in this umbrella review. All meta-analysis were graded against a Measurement Tool to Assess Systematic Reviews 2 (AMSTAR 2) and scored between 8 and 11. Compared to the sham group, the ESWT group showed better results in WOMAC (Western Ontario and McMaster Universities Arthritis Index) [mean difference (MD)=−2.94, 95% CI: −5.52, −0.37, *P*=0.03, I²=60%], Visual Analog Scale (VAS) (MD=−2.0, 95% CI: −2.5, −1.5, *P*<0.01, I²=0%), range of motion (ROM) (MD=17.55, 95% CI: 13.49, 21.61, *P*<0.00001, I²=0%), and Lequesne index (MD=−2.85, 95% CI: −3.64, −2.07, *P*<0.00001, I²=48%).

**Conclusion:**

Based on the results of our analysis, ESWT is now an effective therapy for improving pain and function in patients with KOA.

## Introduction

Highlights

- This study can help clinicians make better decisions.
- Umbrella review represents the pinnacle of evidence-based medicine, serving as the epitome for assessing the quality of meta-analyses, and systematically synthesizing their pertinent findings to generate a comprehensive body of evidence that may offer novel insights for clinical practice.
- Based on the results of our analysis, extracorporeal shock wave therapy is now an effective therapy for improving pain and function in patients with knee osteoarthritis.

Knee osteoarthritis (KOA) is a very common degenerative joint disease and a major contributor to disability. The articular cartilage is primarily affected by degenerative changes and wear, which frequently cause pain, swelling, and restricted mobility^1^. This condition is widespread globally, particularly among the elderly population. According to reports, the incidence of KOA among individuals aged 60 and above is ~50%, and ~80% among those aged 75 and above^2^. KOA imposes various detrimental effects on patients^3^. The most common symptoms include knee joint pain and stiffness. These symptoms can restrict patients’ mobility, impacting their daily activities such as climbing stairs, walking, or standing. Patients may experience swelling and deformity, and the muscles surrounding the joint may undergo atrophy and weakness. The severity of pain and symptoms varies among individuals and may fluctuate during different times and activities^4^.

KOA can be treated with extracorporeal shock wave therapy (ESWT), which is a non-surgical treatment^5^. Non-surgical treatments are becoming increasingly popular in the treatment of knee osteoarthritis, one of which, ESWT, is increasingly being used. ESWT works by delivering high-energy acoustic wave pulses into the patient’s body, targeting the affected area, and stimulating mechanisms such as blood circulation, cellular proliferation and repair, as well as reducing inflammation^6^. These processes facilitate the repair of articular cartilage and alleviate pain in the treated joint. This therapy does not require incisions or pharmacological intervention, making it a non-invasive treatment option. During the specific treatment process, a physician utilizes an external device to guide low-intensity shockwaves to the knee joint area of the patient. Typically, multiple treatment sessions are necessary, with each session lasting ~15–20 min^7^. During the treatment, patients may experience transient mild pain or discomfort, which is generally tolerable. In patients with KOA, extracorporeal shock wave therapy is being used more and more^8–10^. Despite growing interest in the use of ESWT in the treatment of KOA, there are significant research gaps in the comprehensive evaluation of its efficacy and safety. While a number of meta-analyses have been conducted to assess the efficacy of ESWT, there have been inconsistencies in their findings. To address these issues, this study aimed to provide an umbrella review of existing meta-analyses on ESWT treatment of KOA. Conduct a comprehensive assessment of the available evidence and address existing research gaps.

Through this study, patients and clinicians can gain a clearer understanding of the efficacy and safety of ESWT in treating KOA, thereby helping healthcare professionals and patients make informed treatment choices. Umbrella review represents the pinnacle of evidence-based medicine, serving as the epitome for assessing the quality of meta-analyses, and systematically synthesizing their pertinent findings to generate a comprehensive body of evidence that may offer novel insights for clinical practice. The meta-analysis examining the effectiveness of ESWT for KOA were evaluated in the current study. The aim was to assess the consistency of the evidence produced by these meta-analysis and to assess the methodological quality of these meta-analysis. To support the clinical application of ESWT by offering thorough, clear, and precise evidence, in order to aid in KOA’s sane implementation of ESWT.

## Methods

An umbrella review evaluates and compiles data from various meta-analysis on all outcomes^11,12^. In our study, the experimental group was ESWT group and the control group was sham group. We used the procedures outlined in the Cochrane Handbook on conducting umbrella reviews^12–14^. Registered on the PROSPERO website, the work has been reported in line with Preferred Reporting Items for Systematic Reviews and Meta-Analyses (PRISMA), Supplemental Digital Content 1, http://links.lww.com/JS9/B726, Supplemental Digital Content 2, http://links.lww.com/JS9/B727 and A MeaSurement Tool to Assess systematic Reviews 2, (AMSTAR 2), Supplemental Digital Content 3, http://links.lww.com/JS9/B728 Guidelines^15,16^. (Supplementary Material S1, Supplemental Digital Content 4, http://links.lww.com/JS9/B729) Two independent reviewers were assigned to the data retrieval, extraction, processing, and evaluation procedure. In cases of disagreement, a third reviewer intervened and made judgments by comparing their results^17^.

### Search strategy

The search was conducted in three databases, Embase, PubMed, and Web of Science, up until September 2023. The literature retrieval process involved using a combination of subject terms and free words to conduct the search. The English search terms included Extracorporeal, Shock wave Therapies, Osteoarthritides, Meta-analysis, etc. (Supplementary Material S2, Supplemental Digital Content 5, http://links.lww.com/JS9/B730)

### Selection of meta-analysis

One reviewer checked the titles and abstracts in order to weed out any unnecessary ones. The remaining studies’ full-text were located and evaluated separately by two reviewers. The following inclusion criteria were met by the included meta-analysis: (1) They were meta-analysis as defined by the PRISMA^12,18^ and whose intervention must include ESWT. (2) Meta-analysis must comprise at least two trials in their outcome measures, which must be quantitatively synthesized and evaluate either effect or safety. Exclusion criteria: (1) Letters, conference abstracts, protocols, and network meta-analyses are all excluded. (2) Meta-analysis lacking sufficient extractable information about ESWT, such as the number of patients, the number of pooled trials, and the relative effect with 95% CI^17^.

### Data extraction and quality assessment

Two reviewers separately extracted the data and evaluated its quality, while a third reviewer helped to reach a consensus on any differences. Information such as author, year of publication, number of patients, number of original studies, results, average age, sex ratio, etc., were extracted. The Western Ontario and McMaster Universities Arthritis Index (WOMAC), Visual Analog Scale (VAS), Lequesne index, and Range of motion (ROM) were the main outcome measures. Using the AMSTAR 2, two reviewers independently evaluated the methodological quality of the included meta-analysis. When a dispute arises, it will be re-evaluated by the third author to reach a consensus^12,19^. To assess the quality and reliability of the meta-analysis included in this umbrella review, we also used the GRADE (Grading of Recommendations Assessment, Development, and Evaluation) manual evaluation method. GRADE is an evaluation tool widely used in clinical practice guidelines and systematic reviews to assess the quality of evidence and the strength of recommendations. We used GRADE manual evaluation to evaluate measures of study design, risk of bias, consistency of results, indirectness, and uncertainty included in the meta-analysis.

### Statistical analyses

A systematic tool was applied to each eligible meta-analysis to extract the relevant data. Results of eligible meta-analysis were extracted and outcomes were pooled and expressed as mean difference (MD) and risk ratio (RR) with corresponding 95% CI^20^. Using Cochran’s Q statistics and *I* ^*2*^ statistics, the degree of heterogeneity among the included studies that could not be solely ascribed to sampling error was evaluated. The interpretation of *I* ^*2*^ values was as follows: low (*I* ^*2*^: <25%), low to moderate (*I* ^*2*^: 25–50%), moderate to substantial (*I* ^*2*^: 50–75%), or substantial (*I* ^*2*^: >75%)^21^. Additionally, sensitivity analysis was carried out to assess the robustness of the summary estimates and find any particular study that may have significantly contributed to the observed heterogeneity^22^. All meta-analysis were conducted using Review Manager (version 5.4; The Nordic Cochrane Centre, Copenhagen, Denmark) and a two-sided *P* value less than 0.05 was considered statistically significant.

## Results

### Search results

**Figure 1:**
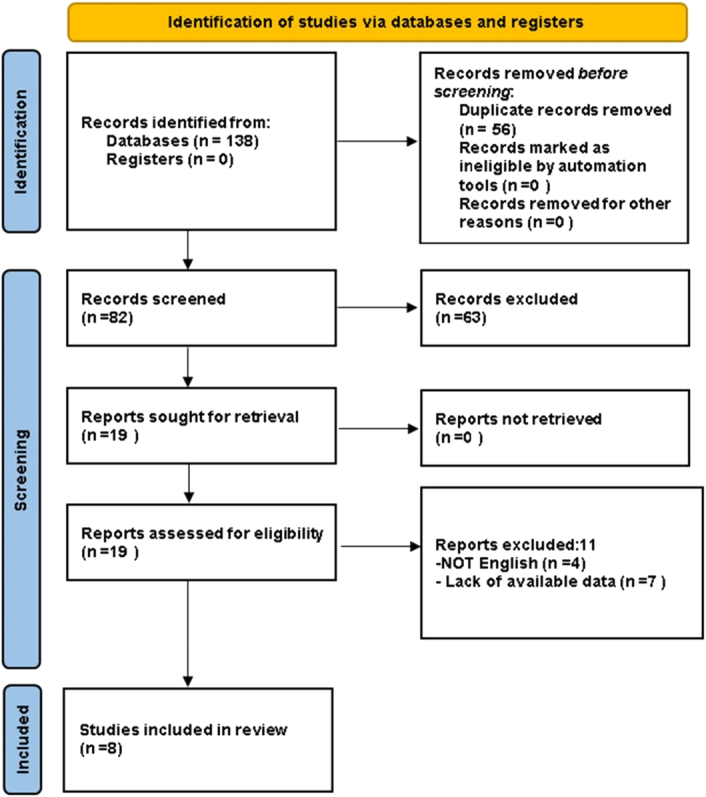
The Preferred Reporting Items for Systematic reviews and Meta-analysis (PRISMA) flow diagram to show study selection.

A total of 138 articles was initially retrieved according to the search strategy, 56 of which were excluded for duplicates, and 63 were excluded by reading the titles and abstracts. Through reading the full text, 11 more studies were excluded (Supplementary material S3, Supplemental Digital Content 6, http://links.lww.com/JS9/B731) Finally, eight studies were included. Figure 1 depicts the literature screening procedure.

### Study characteristics

In this article, the included studies were all published between 2019 and 2023. Table 1 presents basic information about all the included studies. Four meta-analysis included fewer than 10 studies^25–27,30^. Two meta-analysis included over 4000 patients^24,29^. All meta-analysis reported WOMAC scores and VAS scores^23–30^. Three meta-analysis reported the Lequesne index^26,29,30^. Three MAs reported ROM^28–30^. All studies concluded that EWST is effective for KOA. According to Silva *et al.* ^23^, shock wave therapy is believed to improve the function of patients with KOA in the ESWT and alleviate pain at all follow-up time points. According to Wang and colleagues, for up to 12 months following treatment for KOA, ESWT has positive effects on pain relief and physical function. The study also suggests that ESWT treatment has minimal occurrence of complications^25^. According to Ma *et al.* ^26^, In patients with KOA, ESWT is thought to be effective and secure for reducing pain and enhancing knee joint function. According to Hsieh *et al.* ^27^, ESWT can help reduce pain and enhance functional outcomes when used to treat KOA. ESWT is regarded as a successful short-term treatment strategy for reducing pain and restoring function in KOA patients. The study also suggests that ESWT has minimal side effects^28^. According to Li *et al.* ^30^, Physical therapy and a placebo were found to be less effective than ESWT in treating KOA.

**Table 1:** Baseline characteristics of included literatures.

				Total knee replacement cases		Sex ratio				
Study	Year	KL	Total sample size	No. joints in the experimental group	No. joints in the sham group	Man	Woman	Mean age	Outcomes	GRADE
Silva *et al*.^23^	2023	1–3	734	403	331	28%	72%	49.7–72.4	WOMAC, VAS	Low
Oliveira *et al*.^24^	2022	2–3	4798	NA	NA	23%	77%	60±7.3	WOMAC, VAS	Medium
Wang *et al*.^25^	2020	1–3	431	NA	NA	63%	37%	50.9-75	WOMAC, VAS	Medium
Ma *et al*.^26^	2020	NA	589	290	299	37%	63%	63.0	WOMAC, VAS, LI	High
Hsieh *et al*.^27^	2020	1–2	705	322	373	43%	57%	65.4	WOMAC, VAS	Medium
Avendaño-Coy *et al*.^28^	2020	1–4	782	410	467	36%	64%	43–75	WOMAC, VAS, ROM	High
Liao *et al*.^29^	2019	1–3	4844	2604	2240	NA	NA	61.3	WOMAC, VAS, LI, ROM	Medium
Li *et al*.^30^	2019	NA	366	160	197	NA	NA	NA	WOMAC, VAS, LI, ROM	Very Low

KL, Kellgren–Lawreance classification; LI, Lequesne index; NA, not applicable; ROM, range of motion; VAS, Visual Analog Scale; WOMAC, Western Ontario and McMaster Universities Osteoarthritis Index.

### The assessment of meta-analysis

The results of the quality assessment of each study are described in supplementary material S4, Supplemental Digital Content 7, http://links.lww.com/JS9/B732. All of the included studies had AMSTAR 2 scores between 8 and 11, and they were all of moderate to high quality. GRADE evaluation results Only two studies were of low quality overall, and the rest were of medium or high quality.

### Results of meta-analysis

#### Western Ontario and McMaster Universities Arthritis Index

**Figure 2:**
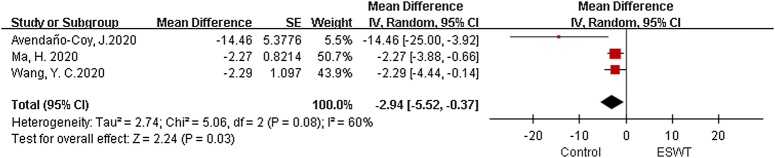
Forest plots of Western Ontario and McMaster Universities Arthritis Index (WOMAC). ESWT, extracorporeal shock wave therapy.

There are 3 meta-analysis, with a total of 1802 patients included^25,26,28^. The analysis conducted using a random effects model revealed statistically significant variations within the ESWT and sham groups (MD=−2.94, 95% CI: −5.52, −0.37, *P*=0.03, I²=60%) (Fig. 2).

#### Visual Analog Scale

**Figure 3:**
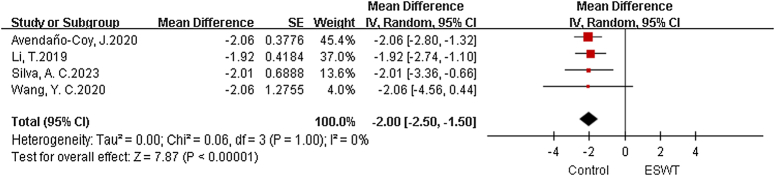
Forest plots of Visual Analog Scale (VAS). ESWT, extracorporeal shock wave therapy.

There are 4 meta-analysis, with a total of 2313 patients included^23,25,28,30^. The analysis conducted using a fixed effects model revealed statistically significant variations within the ESWT and Sham groups. (MD=−2.0, 95% CI: −2.5, −1.5, *P*<0.01, I²=0%) (Fig. 3).

#### Lequesne index

**Figure 4:**
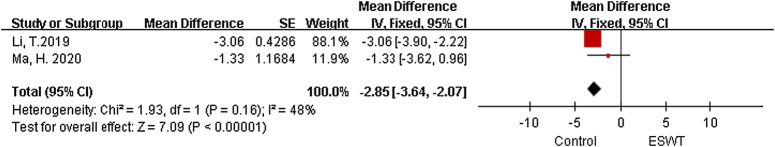
Forest plots of Lequesne index. ESWT, extracorporeal shock wave therapy.

There are two meta-analysis^26,30^ with a total of 15 studies included. The analysis using a fixed effects model showed statistically significant differences between the ESWT and sham groups (MD=−2.85, 95% CI: −3.64, −2.07, *P*<0.00001, I²=48%) (Fig. 4).

#### Range of motion

**Figure 5:**
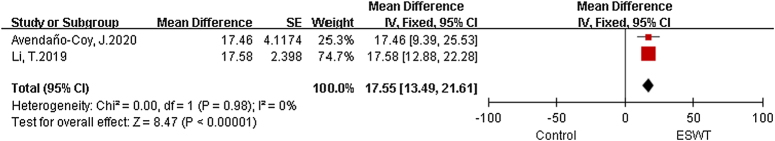
Forest plots of range of motion (ROM). ESWT, extracorporeal shock wave therapy.

There are two meta-analyses^28,30^ with a total of 21 studies included. Between the ESWT and sham groups, the analysis using a fixed effects model found statistically significant differences (MD=17.55, 95% CI: 13.49, 21.61, *P*<0.01, I²=0%) (Fig. 5).

## Discussion

ESWT has been extensively researched for the treatment of KOA in recent times^31–35^. However, the effectiveness of ESWT for KOA remain a matter of debate. The efficacy of ESWT for KOA have not been thoroughly investigated. To evaluate the efficacy of ESWT in treating KOA, we did an umbrella review in this study.

The findings of our study indicate that ESWT can effectively improve VAS scores, WOMAC scores, Lequesne index, and ROM in patients with KOA. There was considerable heterogeneity in the pooled results of WOMAC scores. Sensitivity analysis, after excluding the studies of Avendaño-Coy *et al.* ^28^, revealed an I² value of 0, indicating good homogeneity. This could be due to the relatively small sample sizes of the randomized controlled trials included in this meta-analysis. In addition, due to limitations in the number of included studies, it was not possible to analyze the treatment effects of different energy levels of shock wave therapy separately. Instead, they were combined into one shock wave group, which may introduce bias in the results. Another potential source of heterogeneity could be the significant variability in demographic and clinical characteristics of the included samples. The average age range of the patients included in the studies was quite wide. Furthermore, the duration of symptoms among the included patients ranged from three months to over a year. After excluding the studies that contributed to heterogeneity, the overall results revealed that the ESWT group had superior efficacy compared to the non-ESWT group, with a significant decrease in WOMAC scores after treatment. It is noted that the Lequesne index, VAS score and ROM did not show significant heterogeneity. This indicates that the results of our meta-analysis are very reliable.

ESWT for KOA has been shown to have dose-related effects, with the high-energy group demonstrating larger improvements in pain alleviation and functional results compared to the low-energy group^36^. Of the articles we included, two mentioned that patients receiving high-energy ESWT^36,37^ had greater improvement in VAS scores at two to three months of follow-up than patients receiving low-energy ESWT^36^. These findings imply that high-energy ESWT seems to promote pain alleviation more than low-energy ESWT. The function and pain of individuals with knee OA significantly improved at the majority of follow-up time periods compared to baseline levels, according to Schmitz *et al.*‘s^38^ findings. And that none of the included trials reported any severe adverse effects. These findings concur with what we found. Based on these results, we can conclude that ESWT appears to be an effective treatment for relieving knee OA pain. Wang *et al.* ^25^ discovered that, based on WOMAC and VAS ratings, ESWT effectiveness decreased with time in comparison to baseline values. A number of clinical reasons may lead to decreased efficacy, such as gradual weakening of the effect, which leads to more pain and gradual weakening of the effect, and changes in other treatments. This decline in efficacy can be prevented by repeating ESWT at every time interval or maintaining the same activity despite pain. When assessing the effectiveness of ESWT, adverse reactions are well-known to be a major concern. Therefore, the clinical benefit of using ESWT is diminished if the risk of side effects is high. However, Ma. *et al.* ^26^ showed that ESWT does not increase the risk of local reactions. The safety of ESWT should be further discussed in light of the study by Ma *et al.* ^26^ small sample size.

The mechanism of ESWT in treating KOA has been investigated by researchers. ESWT can enhance tissue repair by activating the body’s biological effects^39^. The application of ESWT can improve local microcirculation by enhancing blood circulation and increasing oxygen supply, thus enhancing tissue nutrition and metabolism, and promoting cartilage and bone tissue repair^40^. ESWT can stimulate cells to release anti-inflammatory cytokines and modulate the balance of inflammatory mediators, thereby attenuating the inflammatory process^41^. ESWT, through its mechanical impact, can disrupt fibrotic tissue and disintegrate calcifications, thereby improving the metabolism and function of cartilage cells and reducing the progression of arthritis inflammation^42^. ESWT stimulates cellular activity, leading to the secretion of synovial fluid by cartilage and synovial cells. The increase in synovial fluid reduces friction between bone and cartilage, thereby alleviating pain and inflammation caused by arthritis^39^. ESWT also stimulates peripheral nerves, leading to the release of neurotransmitters and changes in neural regulation, thus modulating pain perception and nerve function to achieve pain relief in KOA^43^.

The umbrella review has several limitations. (1) Individual SR inherent selection, reporting, and publication bias. (2) Many primary studies did not provide detailed information on follow-up or specific outcome measurements. (3) There are variations in the inclusion/exclusion criteria among the included meta-analysis, which may affect result synthesis. (4) Some key studies are included in multiple meta-analysis. (5) Many meta-analysis only cover patients from specific regions, populations, or conditions, which may limit the generalizability to a broader population. (6) This umbrella review only includes meta-analysis written in English and does not include those written in other languages.

## Conclusion

Based on the results of our analysis, ESWT is now an effective therapy for improving pain and function in patients with KOA.

## Ethical approval

No patients were involved in this study.

## Consent

No patients were involved in this study.

## Source of funding

Science and Technology Innovation Program of Hunan Province (No.2021JJ31105).

## Author contribution

P.T. and W.X. conceived the study. Y.L. and T.W. designed the study. P.T., T.W., and Y.L. undertook the literature review and extracted the data. P.T. and W.X. coded the statistical analysis, fgures, and appendix. P.T. and T.W. interpreted the data and wrote the first draft of the manuscript. All authors read and approved the final manuscript.

## Conflicts of interest disclosure

None.

## Research registration unique identifying number (UIN)

This study has been registered at Prospero.

Registration ID: CRD42023462316.

## Guarantor

Yusheng Li.

## Data availability

The data are publicly available and there are no restrictions.

Data sources: Three databases including PubMed, Embase and Web of science were searched up to August 2023.

Data sharing: The data from this study can be shared with other researchers.

Data processing and analysis: Literature screening, quality evaluation, and data extraction were performed according to inclusion and exclusion criteria. Meta-analysis of outcome indicators was performed using Revman 5.4 software.

Data protection and privacy: The data in this study comply with applicable legal, ethical and privacy regulations.

Repeatability of data: The data in this study are reproducible and robust.

## Provenance and peer review

**P**rovenance and peer review not commissioned, externally peer-reviewed.

## Supplementary Material

SUPPLEMENTARY MATERIAL
