# Supplementary material for: The efficacy of extracorporeal shock wave therapy for knee osteoarthritis : an umbrella review
Source: Int J Surg. 2024 Jan 18;110(4):2389–95. doi: 10.1097/JS9.0000000000001116 (PMC11020044; doi:10.1097/JS9.0000000000001116)
Supplement: SUPPLEMENTARY MATERIAL [file js9-110-2389-s002.docx]

**Identification of studies via databases and registers**

**Records removed *before screening*:**

**Duplicate records removed (n = 56)**

**Records marked as ineligible by automation tools (n =0 )**

**Records removed for other reasons (n =0 )**

**Records identified from:**

**Databases (n = 138)**

**Registers (n = 0)**

**Identification**

**Records screened**

**(n =82)**

**Records excluded**

**(n =63)**

**Reports sought for retrieval**

**(n =19 )**

**Reports not retrieved**

**(n =0 )**

**Screening**

**Reports excluded:11**

**-NOT English (n =4)**

**- Lack of available data (n =7 )**

**Reports assessed for eligibility**

**(n =19 )**

**Studies included in review**

**(n =8)**

**Included**
