# Supplementary material for: The efficacy of extracorporeal shock wave therapy for knee osteoarthritis : an umbrella review
Source: Int J Surg. 2024 Jan 18;110(4):2389–95. doi: 10.1097/JS9.0000000000001116 (PMC11020044; doi:10.1097/JS9.0000000000001116)
Supplement: SUPPLEMENTARY MATERIAL [file js9-110-2389-s005.docx]

## Pubmed

Search: ((((((((((((((((((Extracorporeal Shockwave Therapy[Title/Abstract]) OR (Extracorporeal Shockwave Therapies[Title/Abstract])) OR (Shockwave Therapies, Extracorporeal[Title/Abstract])) OR (Shockwave Therapy, Extracorporeal[Title/Abstract])) OR (Therapy, Extracorporeal Shockwave[Title/Abstract])) OR (Shock Wave Therapy[Title/Abstract])) OR (Shock Wave Therapies[Title/Abstract])) OR (Therapy, Shock Wave[Title/Abstract])) OR (Extracorporeal Shock Wave Therapy[Title/Abstract])) OR (Extracorporeal High-Intensity Focused Ultrasound Therapy[Title/Abstract])) OR (Extracorporeal High Intensity Focused Ultrasound Therapy[Title/Abstract])) OR (HIFU Therapy[Title/Abstract])) OR (HIFU Therapies[Title/Abstract])) OR (Therapy, HIFU[Title/Abstract])) OR (High-Intensity Focused Ultrasound Therapy[Title/Abstract])) OR (High Intensity Focused Ultrasound Therapy[Title/Abstract])) OR ("Extracorporeal Shockwave Therapy"[Mesh])) AND ((((((((((((Osteoarthritis[Title/Abstract]) OR (Osteoarthritides[Title/Abstract])) OR (Osteoarthrosis[Title/Abstract])) OR (Osteoarthroses[Title/Abstract])) OR (Arthritis, Degenerative[Title/Abstract])) OR (Arthritides, Degenerative[Title/Abstract])) OR (Degenerative Arthritides[Title/Abstract])) OR (Degenerative Arthritis[Title/Abstract])) OR (Arthrosis[Title/Abstract])) OR (Arthroses[Title/Abstract])) OR (Osteoarthrosis Deformans[Title/Abstract])) OR ("Osteoarthritis"[Mesh]))) AND (((meta-analysis[Title/Abstract]) OR (systematic review[Title/Abstract])) OR (("Systematic Reviews as Topic"[Mesh]) OR (meta-analysis[MeSH Terms]))) Sort by: Journal

## Web of Science Search Strategy

1: (((((((((((((((TS=(Extracorporeal Shockwave Therapy)) OR TS=(Extracorporeal Shockwave Therapies)) OR TS=(Shockwave Therapies, Extracorporeal)) OR TS=(Shockwave Therapy, Extracorporeal)) OR TS=(Therapy, Extracorporeal Shockwave)) OR TS=(Shock Wave Therapy)) OR TS=(Shock Wave Therapies)) OR TS=(Therapy, Shock Wave)) OR TS=(Extracorporeal Shock Wave Therapy)) OR TS=(Extracorporeal High-Intensity Focused Ultrasound Therapy)) OR TS=(Extracorporeal High Intensity Focused Ultrasound Therapy)) OR TS=(HIFU Therapy)) OR TS=(HIFU Therapies)) OR TS=(Therapy, HIFU)) OR TS=(High-Intensity Focused Ultrasound Therapy)) OR TS=(High Intensity Focused Ultrasound Therapy) and Preprint Citation Index (Exclude – Database)

2: ((((((((((TS=(Osteoarthritis)) OR TS=(Osteoarthritides)) OR TS=(Osteoarthrosis)) OR TS=(Osteoarthroses)) OR TS=(Arthritis, Degenerative)) OR TS=(Arthritides, Degenerative)) OR TS=(Degenerative Arthritides)) OR TS=(Degenerative Arthritis)) OR TS=(Arthrosis)) OR TS=(Arthroses)) OR TS=(Osteoarthrosis Deformans) and Preprint Citation Index (Exclude – Database)

3: (TS=(Systematic review)) OR TS=(Meta-analysis) and Preprint Citation Index (Exclude – Database)

4: #1 AND #2 AND #3 and Preprint Citation Index (Exclude – Database)

## Embase

No. Query Results

#11. #8 AND #9 AND #10

#10. #2 OR #5

#9. #3 OR #4 OR #7

#7. 'systematic review':ab,ti OR 'meta

analysis':ab,ti

#6. osteoarthritis:ab,ti OR osteoarthritides:ab,ti OR

osteoarthrosis:ab,ti OR osteoarthroses:ab,ti OR

'arthritis, degenerative':ab,ti OR 'arthritides,

degenerative':ab,ti OR 'degenerative

arthritides':ab,ti OR 'degenerative

arthritis':ab,ti OR arthrosis:ab,ti OR

arthroses:ab,ti OR 'osteoarthrosis

deformans':ab,ti

#5. 'extracorporeal shockwave therapy':ab,ti OR

'extracorporeal shockwave therapies':ab,ti OR

'shockwave therapies, extracorporeal':ab,ti OR

'shockwave therapy, extracorporeal':ab,ti OR

'therapy, extracorporeal shockwave':ab,ti OR

'shock wave therapy':ab,ti OR 'shock wave

therapies':ab,ti OR 'therapy, shock wave':ab,ti

OR 'extracorporeal shock wave therapy':ab,ti OR

'extracorporeal high-intensity focused ultrasound

therapy':ab,ti OR 'extracorporeal high intensity

focused ultrasound therapy':ab,ti OR 'hifu

therapy':ab,ti OR 'hifu therapies':ab,ti OR

'therapy, hifu':ab,ti OR 'high-intensity focused

ultrasound therapy':ab,ti OR 'high intensity

focused ultrasound therapy':ab,ti

#4. 'meta analysis'/exp OR 'meta analysis'

#3. 'systematic review'/exp OR 'systematic review'

#2. 'shock wave therapy'/exp OR 'shock wave therapy'

#1. 'osteoarthritis'/exp OR 'osteoarthritis'
