# Supplementary material for: The efficacy of extracorporeal shock wave therapy for knee osteoarthritis : an umbrella review
Source: Int J Surg. 2024 Jan 18;110(4):2389–95. doi: 10.1097/JS9.0000000000001116 (PMC11020044; doi:10.1097/JS9.0000000000001116)
Supplement: SUPPLEMENTARY MATERIAL [file js9-110-2389-s006.docx]

# Excluded studies and their reasons：

| Study | Reason for exclusion |
| --- | --- |
| (1) | NOT English |
| (2) | NOT English |
| (3) | NOT English |
| (4) | NOT English |
| (5) | Lack of available data |
| (6) | Lack of available data |
| (7) | Lack of available data |
| (8) | Lack of available data |
| (9) | Lack of available data |
| (10) | Lack of available data |
| (11) | Lack of available data |

# References

1. Gu J, Li K, Zhang Q, Li L, Bai Z, Wang S. Clinical Efficacy of Extracorporeal Shock Wave in the Treatment of Knee Osteoarthritis: A Meta-analysis. Rehabilitation Medicine. 2022;32(4):359-66.

2. Huangfu Z, Wei D, Ao Y. Systematic evaluation and meta-analysis of extracorporeal shock wave therapy in the treatment of knee osteoarthritis. Chinese Journal of Tissue Engineering Research. 2020;24(27):4414-20.

3. Huang DC, Wang ZK, Cao XW. Comparison of the short-term efficacy of extracorporeal shock wave therapy for middle-aged and elderly knee osteoarthritis: A meta-analysis. Chinese Journal of Tissue Engineering Research. 2020;25(9):1471-6.

4. Chen L, Ye L, Liu H, Yang P, Yang B, Seixas A. Extracorporeal Shock Wave Therapy for the Treatment of Osteoarthritis: A Systematic Review and Meta-Analysis. Chinese Journal of Tissue Engineering Research. 2020;2020.

5. Häußer J, Wieber J, Catalá-Lehnen P. The use of extracorporeal shock wave therapy for the treatment of bone marrow oedema - a systematic review and meta-analysis. Journal of orthopaedic surgery and research. 2021;16(1):369.

6. Tai TW, Hsieh CK, Chang J, Liu ZW. Extracorporeal shockwave therapy to treat osteoarthritis of knees: A meta-analysis. Osteoporosis International. 2020;31(SUPPL 1):S418.

7. Al-Abbad H, Allen S, Morris S, Reznik J, Biros E, Paulik B, et al. The effects of shockwave therapy on musculoskeletal conditions based on changes in imaging: A systematic review and meta-analysis with meta-regression. BMC Musculoskeletal Disorders. 2020;21(1).

8. Tang HY, Zhao Y, Li YZ, Wang TS. Effectiveness of extracorporeal shock wave monotherapy for avascular necrosis of femoral head: A systematic review protocol of randomized controlled trial. Medicine (Baltimore). 2019;98(14):e15119.

9. Ferreira RM, Torres RT, Duarte JA, Gonçalves RS. Non-Pharmacological and Non-Surgical Interventions for Knee Osteoarthritis: A Systematic Review and Meta-Analysis. Acta Reumatol Port. 2019;44(3):173-217.

10. Liao C-D, Tsauo J-Y, Chen H-C, Liou T-H. Efficacy of Extracorporeal Shock Wave Therapy for Lower-Limb Tendinopathy: A Meta-analysis of Randomized Controlled Trials. American Journal of Physical Medicine & Rehabilitation. 2018;97(9):605-19.

11. Zhang Q, Liu L, Sun W, Gao F, Cheng L, Li Z. Extracorporeal shockwave therapy in osteonecrosis of femoral head a systematic review of now available clinical evidences. Medicine (United States). 2017;96(4).
