# Supplementary material for: The efficacy of extracorporeal shock wave therapy for knee osteoarthritis : an umbrella review
Source: Int J Surg. 2024 Jan 18;110(4):2389–95. doi: 10.1097/JS9.0000000000001116 (PMC11020044; doi:10.1097/JS9.0000000000001116)
Supplement: SUPPLEMENTARY MATERIAL [file js9-110-2389-s007.docx]

**Table 1. Quality evaluation of included studies.**

| Study | 1. | 2. | 3. | 4. | 5. | 6. | 7. | 8. | 9. | 10. | 11. | Total  score |
| --- | --- | --- | --- | --- | --- | --- | --- | --- | --- | --- | --- | --- |
| Silva, A. C. 2023[[1](#_ENREF_1)] | Y | Y | Y | Y | Y | Y | Y | Y | Y | N | Y | 9 |
| Oliveira, S. 2022[[2](#_ENREF_2)] | Y | Y | Y | N | N | Y | Y | Y | Y | Y | Y | 9 |
| Wang, Y. C. 2020[[3](#_ENREF_3)] | Y | Y | Y | Y | Y | Y | Y | Y | Y | N | Y | 10 |
| Ma, H. 2020[[4](#_ENREF_4)] | Y | Y | Y | Y | Y | Y | Y | N | Y | Y | Y | 11 |
| Hsieh, C. K. 2020[[5](#_ENREF_5)] | Y | Y | Y | Y | N | Y | Y | Y | Y | Y | Y | 10 |
| Avendaño-Coy, J. 2020[[6](#_ENREF_6)] | Y | Y | Y | Y | Y | Y | Y | Y | Y | Y | Y | 11 |
| Liao, C. D. 2019[[7](#_ENREF_7)] | Y | Y | Y | N | Y | Y | Y | Y | Y | Y | Y | 10 |
| Li, T. 2019[[8](#_ENREF_8)] | Y | Y | Y | N | Y | N | Y | Y | Y | Y | Y | 8 |

Scale of item score: N, no; Y, yes.

The AMSTAR criteria are (1) a priori design; (2) duplicate study selection and data extraction; (3) comprehensive literature search; (4) inclusive publication status; (5) included studies provided; (6) characteristics of included studies provided; (7) quality assessment of studies; (8) study quality used appropriately in formulating conclusions; (9) appropriate methods used to combine studies; (10) publication bias assessed; and (11) conflict of interest stated.

**Table 2. GRADE evaluation of included studies.**

| Study | Research design | Bias risk | Result consistency | Indirectness | | Uncertainty | Overall quality |
| --- | --- | --- | --- | --- | --- | --- | --- |
| Silva, A. C. 2023[[1](#_ENREF_1)] | low | low | high | medium | low | | Low |
| Oliveira, S. 2022[[2](#_ENREF_2)] | high | medium | high | medium | low | | Medium |
| Wang, Y. C. 2020[[3](#_ENREF_3)] | high | low | medium | low | medium | | Medium |
| Ma, H. 2020[[4](#_ENREF_4)] | high | low | high | low | low | | High |
| Hsieh, C. K. 2020[[5](#_ENREF_5)] | high | low | high | medium | medium | | Medium |
| Avendaño-Coy, J. 2020[[6](#_ENREF_6)] | high | low | high | low | low | | High |
| Liao, C. D. 2019[[7](#_ENREF_7)] | high | medium | Low | low | low | | Medium |
| Li, T. 2019[[8](#_ENREF_8)] | low | low | medium | high | low | | Very Low |

# References

1. Silva AC, Almeida VS, Veras PM, et al. Effect of extracorporeal shock wave therapy on pain and function in patients with knee osteoarthritis: a systematic review with meta-analysis and grade recommendations. Clinical rehabilitation 2023;**37**(6):760-73 doi: 10.1177/02692155221146086.

2. Oliveira S, Andrade R, Valente C, et al. Mechanical-based therapies may reduce pain and disability in some patients with knee osteoarthritis: A systematic review with meta-analysis. Knee 2022;**37**:28-46 doi: 10.1016/j.knee.2022.05.005.

3. Wang YC, Huang HT, Huang PJ, Liu ZM, Shih CL. Efficacy and safety of extracorporeal shockwave therapy for treatment of knee osteoarthritis: A systematic review and meta-analysis. Pain Medicine (United States) 2020;**21**(4):822-35 doi: 10.1093/PM/PNZ262.

4. Ma H, Zhang W, Shi J, Zhou D, Wang J. The efficacy and safety of extracorporeal shockwave therapy in knee osteoarthritis: A systematic review and meta-analysis. International Journal of Surgery 2020;**75**:24-34 doi: 10.1016/j.ijsu.2020.01.017.

5. Hsieh CK, Chang CJ, Liu ZW, Tai TW. Extracorporeal shockwave therapy for the treatment of knee osteoarthritis: a meta-analysis. International Orthopaedics 2020;**44**(5):877-84 doi: 10.1007/s00264-020-04489-x.

6. Avendaño-Coy J, Comino-Suárez N, Grande-Muñoz J, Avendaño-López C, Gómez-Soriano J. Extracorporeal shockwave therapy improves pain and function in subjects with knee osteoarthritis: A systematic review and meta-analysis of randomized clinical trials. International Journal of Surgery 2020;**82**:64-75 doi: 10.1016/j.ijsu.2020.07.055.

7. Liao CD, Tsauo JY, Liou TH, Chen HC, Huang SW. Clinical efficacy of extracorporeal shockwave therapy for knee osteoarthritis: a systematic review and meta-regression of randomized controlled trials. Clinical rehabilitation 2019;**33**(9):1419-30 doi: 10.1177/0269215519846942.

8. Li T, Ma J, Zhao T, Gao F, Sun W. Application and efficacy of extracorporeal shockwave treatment for knee osteoarthritis: A systematic review and meta-analysis. Experimental and Therapeutic Medicine 2019;**18**(4):2843-50 doi: 10.3892/etm.2019.7897.
